# Therapeutic options in pediatric non alcoholic fatty liver disease: current status and future directions

**DOI:** 10.1186/1824-7288-38-55

**Published:** 2012-10-17

**Authors:** Pietro Vajro, Selvaggia Lenta, Claudio Pignata, Mariacarolina Salerno, Roberta D’Aniello, Ida De Micco, Giulia Paolella, Giancarlo Parenti

**Affiliations:** 1 Chair of Pediatrics, Medical School of the University of Salerno, Salerno, Italy, and ELFID, Naples, Italy; 2 Department of Pediatrics, University of Naples “Federico II”, Naples, Italy

**Keywords:** Non alcoholic fatty liver disease, Children, Therapy

## Abstract

The epidemics of overweight and obesity has resulted in a significant increase of non alcoholic fatty liver disease (NAFLD), a potentially progressive condition. Currently, obesity related hepatopathy represents therefore the main cause of pediatric chronic liver disease. The first choice treatment at all ages is weight loss and/or lifestyle changes, however compliance is very poor and a pharmacological approach has become necessary. In the present article we present a systematic literature review focusing on established pediatric NALFD drugs (ursodeoxycholic acid, insulin sensitizers, and antioxidants) and on innovative therapeutic options as well.

Regarding the former ones, a pediatric pilot study highlighted that ursodeoxycholic acid is not efficient on transaminases levels and bright liver. Similarly, a recent large scale, multicenter randomized clinical trial (TONIC study) showed that also insulin sensitizers and antioxidant vitamin E have scarce effects on serum transaminase levels. Among a large series of novel therapeutic approaches acting on recently proposed different pathomechanisms, probiotics seem hitherto the most interesting and reasonable option for their safety and tolerability. Toll-like receptors modifiers, Pentoxifylline, and Farnesoid X receptors agonists have been still poorly investigated, and will need further studies before becoming possible promising innovative therapeutic strategies.

## Introduction

**Figure 1:**
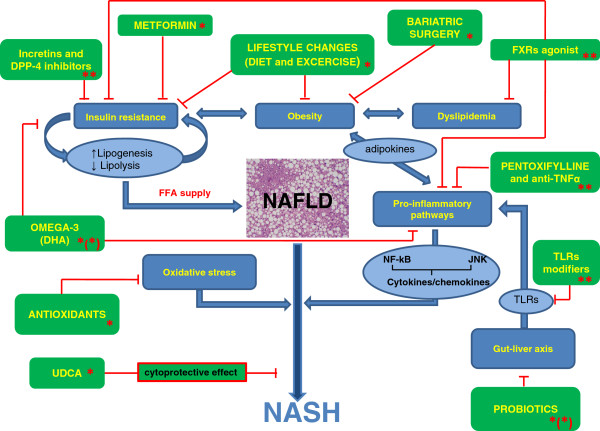
**NAFLD pathogenesis-driven old and novel potential treatments in pediatric NAFLD/NASH.** Symbols: * current , ** potential, and/or *(*) partially established therapeutic approaches in pediatric NAFLD. ABBREVIATIONS DHA, docosahexanoic acid; DPP-4, dipeptidyl peptidase-4; FFA, free fatty acid; FXR, farnesoid X receptor; NAFLD, non alcoholic fatty liver disease; NASH, non alcoholic steatohepatitis; TLR, toll-like receptor; UDCA, ursodeoxycholic acid.

Non alcoholic fatty liver disease is nowadays the most common cause of chronic liver disease also in pediatric age, as a result of the increasing prevalence of childhood obesity. It represents a spectrum of liver diseases ranging from simple steatosis to steatohepatitis, with, in some cases, possible fibro/cirrhotic progression, thus increasing liver-related morbidity and mortality [1]. The mainstay of NAFLD therapy is represented by lifestyle interventions on obesogenic environment and sedentary life, which aim to improve obesity, obesity-related hepatic changes, and quality of life as well [2]. Unfortunately this target is difficult to be achieved, and results are unsatisfactory. In fact, only a little proportion of individuals is really able to steadily lose weight and to practice physical exercises [1]. No agreement exists on NAFLD management of obese children who result not-compliant to prescriptions. Since several established pathogenetic mechanisms (in particular insulin resistance, oxidative stress, and apoptosis) seem to be involved in NAFLD, a number of therapeutic agents targeting these mechanisms (Figure 1) have been tested not only in animal models but also in human adults and in children. Results however are still puzzling and/or unsatisfactory [1,3].

The aim of this article is to review current NAFLD treatments and possible novel options in the pediatric age group, by providing recent evidences from literature.

## Materials and methods

### Search strategy and inclusion criteria

Through the MEDLINE database we searched for articles on NAFLD treatment in pediatric age appearing from 1^st^ May 2009 (date of publication of the meta-analysis of Socha et al. [3]) to 1^st^ August 2012. No language restrictions were imposed. Eligible studies and research articles on NAFLD therapy in pediatric age were reviewed. When paucity/absence of pediatric data were evident we retrieved also articles on NAFLD therapy in adults. The electronic literature search was performed using the following keywords which had been suggested by previous manual search: NAFLD, NASH, fatty liver, treatment and/or therapy, lifestyle interventions, weight loss, physical activity, antioxidant and/or vitamin E, metformin and/or insulin sesitizers, ursodeoxycholic acid, probiotics and prebiotics, omega-3-fatty acid and/or docosahexaenoic acid, bariatric surgery, toll like receptors modifiers, pentoxifylline, farnesoid X receptor agonist, incretin mimetics, dipeptidyl peptidase-4 inhibitors, angiotensin-converting enzyme (ACE) inhibitors, angiotensin-receptor blockers (ARBs), children, adults, animal model. Title and abstract of each retrieved reference was screened by one author. Full papers were screened independently by two researchers. Discrepancies between reviewers were resolved by consensus. A narrative review of novel and old therapeutic strategies was conducted.

### Data extraction

Data were collected by using a data extraction form. Studies were collected for old/present and novel/potential pharmacological strategies both in children and adults and/or in animal models.

## Results

### Lifestyle interventions

#### Weight loss

Weight reduction in NAFLD patients reduces the delivery of free fatty acids (FFA) to the liver, improving extra-hepatic insulin sensitivity by means of a better peripheral glucose utilization. Furthermore it promotes a reduction of reactive oxygen substances (ROS) and adipose tissue inflammation [4].

Recent studies confirmed an amelioration in transaminases levels and in several metabolic parameters (lipids, fasting glucose and insulin sensitivity indices) also in pediatric age [5-8].

A sucrose-rich diet (e.g. soft-drinks) increases the hepatic synthesis of triglycerides: rats and humans that are fed either sucrose or fructose enriched diets develop fatty and fibrotic liver. Fructose reduction may decrease insulin resistance (IR) and lipogenesis, and also hepatic pro-inflammatory/fibrogenetic effects [9].

Other helpful proposed dietetic measures include the reduction of dietary intake of satured/trans fat, increased intake of fibers [10] and a higher intake of polyunsaturated fat (omega-3) [11].

Weight loss (target= approximately 5-10% of basal weight) should be achieved gradually, since extreme slimming diets may lead to the onset of severe metabolic disorders and promote liver histological damage. Dietary interventions are still not based on precise evidence-based guidelines: in childhood however diets must be balanced to allow a healthy and harmonic growth [12]. The ESPGHAN Committee on Nutrition [13] suggests that energy intake should be individually determined, and slowly rather than rapidly absorbed carbohydrates should be preferred. A clinical approach in older children should consist in a weight loss of about 500 g / week. In younger children one might suggest even to simply not gain further weight in order to improve the weight-height ratio, and also to improve waist circumference (i.e. the most sensitive marker of visceral obesity and its related liver involvement) [7,14].

#### Physical activity

Physical activity should be integrated in NAFLD therapy because of its beneficial effect independent of weight loss, by enhancing insulin sensitivity and glucose homeostasis [15]. Exercise has a beneficial effect also on FFA metabolism, by enhancing whole-body lipid oxidation [16] and decreasing hepatic triglyceride accumulation [17].

In obese adolescents a three-month resistance exercise program, which involved all major muscle groups 2 × 1 h/week, resulted in significant strength and lean body mass gain. Although hepatic fat content remained fairly stable, hepatic insulin sensitivity increased and glucose production rate decreased, without weight loss [18]. Despite aerobic exercise seems to have more extensive effects, a longer duration and/or a more intensive resistance exercise program may be required for reduction of hepatic fat content. For those who have physical limitations and/or low motivation that prevent them from performance of aerobic activity, resistance exercise can serve as an alternative option.

Lifestyle changes should include all family members to increase compliance, along with a multidisciplinary approach, including childhood dedicated dietician and psychologist. Pediatricians and pediatric gastroentero-hepatologists should have a leading role in the case management of pediatric NAFLD, working with other professional specialties involved in physical and intellectual growth [9].

Since treatment options are nowadays still limited, obesity prevention should still be the optimal strategy in the management of NAFLD. In view of the poor adherence to diets and exercise, novel strategies have also been envisaged, including “e-health platforms”. These are aimed at improving treatment compliance and promoting the mechanisms of patients self-control, to obtain weight loss maintenance, and to prevent relapse by establishing healthy lifestyle habits [19].

### Drug treatments for NAFLD in pediatric age

When diet and/or exercise are not obtainable, a pharmacological approach acting upon one or more specific targets involved in NAFLD aetiopathogenesis should be used (Figure 1). Insulin sensitizers, antioxidants and cytoprotective agents are the drugs which have hitherto been more extensively used.

### Insulin sensitizers

Hepatic insulin resistance has been highlighted in most of the children/adolescents with NAFLD [20]. Even if the molecular mechanism is poorly understood, insulin resistance has a key role in NAFLD by promoting the storage of FFA. For this reason it might be considered an advantageous therapeutic target [1]. The only insulin-sensitizer which has been well evaluated in pediatric NAFLD is metformin. A pilot study in 10 non diabetic children with NASH showed improvement of fatty liver at Magnetic Resonance Spectroscopy, and reduction of ALT level [21]. Subsequent studies however yielded divergent results [22]. The most recent TONIC study [a large, multicenter RCT evaluating the effect of a 2 year therapy with Vitamin E (400 IU twice daily) or insulin sensitizer metformin (500 mg twice daily) or placebo in 173 children with biopsy-confirmed NAFLD] substantiated its scarce effectiveness in lowering serum ALT, with only marginal effects on hepatic histology [23].

### Antioxidants

Oxidative stress is considered another major contributor to the progression from simple steatosis to NASH by protecting susceptible components of biological membranes from lipid peroxidation. Alpha-tocopherol (vitamin E) is the more extensively studied antioxidant in pediatric NAFLD. In an open label pediatric trial Lavine et colleagues found that Vitamin E (400–1.200 IU/day) may induce a decrease of serum transaminases levels, which was not related to a reduction in bright liver at ultrasonography and BMI values [24]. Other studies comparing the effect of Vitamin E [25,26] ± ascorbic acid [27] vs. weight loss alone, however showed that antioxidant treatment was not more efficient than exclusive lifestyle changes. In the double blind RCT study TONIC [23], Vitamin E treatment did not attain a significant and sustained decrease in ALT levels compared to placebo. However it was better than placebo in inducing resolution of an histologically borderline or defined NASH, and in improving hepatocellular ballooning and NAFLD activity histological score. Cysteamine bitartrate is a recently described potent antioxidant which has been hitherto used only in a small pediatric NAFLD pilot study with promising effects on reduction of transaminase levels [28]. It is currently under evaluation in a multicenter placebo-controlled clinical trial (Cysteamine Bitartrate Delayed-Release for the Treatment of NAFLD in Children) [29].

Other newer antioxidants have shown to represent potential therapeutic tools but have been studied only in adult NAFLD [1].

### Hepatoprotective agents

The hydrophilic bile acid Ursodeoxycholic acid (UDCA) might antagonize the progression of NAFLD/NASH, through protection of hepatocytes from bile salts-mediated mitochondrial injury, antiapoptotic signaling pathway, and immunomodulatory function [30]. However, conventional doses of UDCA used in a pilot pediatric RCT were not effective on ALT levels and ultrasonographic liver abnormalities [31]. These data have been confirmed also in several adults studies [32]. Presently it is under verification if (potentially risky [33]) higher doses of UDCA may be necessary [1].

### Innovative drugs

Summing up, ordinary NAFLD therapeutic approaches reviewed above appear most often unsatisfactory or inadequate both in adults and in children. Table 1 summarizes a series of newer treatment targets which have been partially explored in preliminary animal and/or patients studies and may therefore deserve attention for more extensive upcoming investigation in adult and pediatric NAFLD as well.

**Table 1:** Still not conventional NAFLD treatment targets

**Therapeutic target**	**Pharmacolological treatment**
Gut-liver axis	Probiotics [34]
Dyslipidemia/Insulin Resistance	Omega-3 DHA (docosahexaenoic acid) [12]
TNF-α pathway	Pentoxifylline and anti-TNF-α [35]
FXRs pathway	Agonist of the farnesoid X receptor (FXR) [36]

### Probiotics and prebiotics

A growing body of evidence [37] shows that the gut microbiota controls obesity and visceral fat storage. Specific variations in gut microbiota in early life may determine a major risk factor of obesity and its complications later in life [38]. Small intestinal bacterial overgrowth (SIBO) (a frequent condition in obese individuals, mainly prompted by slowing of the oro-coecal transit time) may promote NAFLD progression to non-alcoholic steatohepatitis by enhancing intestinal permeability and by favoring absorption of endotoxins with pro-inflammatory and pro-fibrogenetic effects on the liver [39].

Probiotics are live microorganisms which when consumed in adequate amounts, confer an healthy benefit to the host [40]. Gut microbiota manipulation with probiotics in rodents with fatty liver reduces intestinal inflammation and improves the epithelial barrier function [41,42]. Therefore, probiotics could represent a new effective treatment also in NAFLD human patients. Loguercio and colleagues have shown that probiotics may reduce NAFLD liver injury and may improve liver function tests [43]. The Cochrane review [44] and a subsequent pediatric meta-analysis [3] however have highlighted that probiotics treatment in patients with NAFLD and non alcoholic steatohepatitis could not be recommended because of the lack of randomized clinical trials. More recently a double-blind RCT showed that the treatment with 500 million of Lactobacillus bulgaricus and Streptococcus thermophiles/day in adults with biopsy-proven NAFLD causes a reduction of liver transaminases level [45]. In children, another double-blind RCT study also showed that obese patients with NAFLD (mean age 10.7 years), treated with Lactobacillus GG (12 billion CFU/day for 8 weeks), irrespective of changes in BMI z score and visceral fat, reached a significant decrease (up to normalization in 80% of cases) in alanine aminotransferase and in antipeptidoglycan-polysaccharide antibodies (a SIBO marker). TNF-α serum levels, and bright liver parameters remained quite stable [34].

Prebiotics are non digestible food ingredients that beneficially affect the host by selectively stimulating growth and/or modifying metabolic activity of selected intestinal bacteria [40]. By reducing the risk of obesity and altering the composition of gut microbiota, prebiotics may represent therefore another potential therapeutic approach. Studies in obese rats have shown that prebiotic fibers improve or normalize gut microbiota dysbiosis by increasing Firmicutes and decreasing Bacteroidetes phylae [46]. Gut microbiota modulation is correlated with an improvement of glucose, energy intake, insulin, satiety hormones, hepatic cholesterol, and triglyceride accumulation [45].

### Omega-3 long chain polyunsaturated fatty acids

Polyunsaturated fatty acids (PUFAs) class is present in several natural foods and includes “essential fatty acids” like ω-3 and ω-6 acids that the body needs but cannot produce. The balance between dietary ω-3 and ω-6 strongly affects several functions of PUFAs. Recent pharmacological studies in NAFLD animal models and in adult humans focusing on the effect of oral treatment with ω-3 fatty acids, demonstrate that they have both anti-inflammatory and insulin sensitizing properties, suggesting a potential role in treatment of NAFLD [47]. In NAFLD children ω3-docosahexaenoic acid treatment for 6 months improved ultrasonographic fatty liver and insulin sensitivity [12]. Because this treatment is well tolerated in pediatric population, DHA deserve further studies in the management of children with NAFLD.

### A surgical approach: bariatric surgery

Bariatric surgery may be proposed for a durable weight loss in individuals with severe obesity. A study in young adults (median age = 18.6 years) showed that bariatric surgery, by causing significant weight loss, leads to an improvement in clinical and hepatic histological parameters [48]. The guidelines for younger ages however are not yet fully standardized, and studies on safety and long term efficacy are therefore needed in developing and transition age [49].

### Other potential novel candidate therapeutic targets

A series of other interesting approaches, hitherto explored only in NAFLD animal models or in few pilot studies in adults will possibly become in future the object of study in pediatric population, as well (Table 1).

### TNF-α pathway antagonists

TNF-α and other adipocytokines produced by adipose tissue are involved in NAFLD progression. Pentoxifylline, a phosphodiesterase inhibitor, exerts immunomodulatory functions by antagonizing the TNF-α pathway. In adults with NASH, pentoxifylline treatment showed good tolerability and could decrease serum ALT levels and improve histological features, as well [35].

### Farnesoid X receptor (FXR) agonist

The nuclear bile acid receptor FXR, strongly expressed in bowel and liver, is probably involved in NAFLD pathogenesis, by mediating control of lipids and glucose homeostasis, and controlling bacterial flora growth. Altogether, these effects may induce reduction of hepatic inflammation and fibrogenesis, through different mechanisms. Therefore, recently developed FXR agonists have a potential role in the pharmacological therapy of NAFLD/NASH [36].

### Toll-like receptor (TLR) modifiers

TLRs are receptors sensing microbial components of gut microbiota. A number of recent evidences suggests the role of SIBO and increased intestinal permeability in NAFLD, by exposing -via portal vein- the liver to an high load of intestinal noxae including lipopolysaccharide and other pathogen-associated molecular patterns [50]. Furthermore, TLRs stimulation causes downstream activation of the inflammatory response. Pro-inflammatory patterns result in production of cytokines and chemokines implicated in progression from simple steatosis to steatohepatitis and fibro-cirrhosis; so therapeutic manipulation of innate immune system through TLRs modifiers, formerly evaluated for autoimmune diseases [51], might be a new potential therapeutic target for pediatric NAFLD, but further studies are necessary.

### Incretin mimetics and DPP-4 (dipeptidyl peptidase-4) inhibitors

Glucagon-like peptid-1 (GLP-1) is an incretin secreted in response to food intake, allotted to multiple functions, including, the stimulation of glucose-dependent insulin secretion and inhibition of glucagon release. The enzyme DPP-4 rapidly degrades circulating GLP-1 (half-life: 1–2 minutes). Recent animal model and NAFLD adults studies showed an effective role of GLP-1 receptor agonists resistent to DPP-4 (such as exenatide and liraglutide) or DPP-4 inhibitors (e.g. some gliptins) as a promising new therapy in NAFLD for their ability in modulating fatty acid oxidation, decreasing lipogenesis, and improving hepatic glucose metabolism [52].

### Other pharmacological agents

There are not evidences about the rational for the use of lipid-lowering agents, angiotensin-converting enzyme (ACE) inhibitors, and angiotensin-receptor blockers (ARBs) in treatment of pediatric NAFLD [1].

## Discussions

Data here reviewed confirm that weight loss and/or lifestyle changes remain the first choice treatment for adult and pediatric NAFLD, as well. Management strategy for an obese child with NAFLD should try to attain a gradual weight reduction by a multidisciplinary and long term therapeutic program combining diet and physical exercise. Diet prescription should be part of a well-structured family-based program to enhance self-motivation and success with diet. Physical exercise should be encouraged at all levels, since it might allow better metabolic control, weight reduction or even only its simple stability. Lack of compliance however remains a relevant problem, and dietary therapy for obesity generally fails to achieve its desired target. There is mounting indication that the high rate of relapse from weight loss during dietary therapy occurs because of complex compensatory biological adaptations and is not caused simply by lack of discipline and will power [2].

Disappointing results obtained also by the current standard pharmacological treatments (antioxidants and insulin-sensitizers) in the large multicenter TONIC study [23] call for careful re-thinking on the strategies hitherto pursued for the treatment of NAFLD, both in adults and in children. Future studies will probably need resorting to combined tailored treatments, targeting more than one pathogenetic mechanism.

The recent growing body of evidence on the role of gut microbiota and gut-liver-axis both in provoking or worsening obesity itself and/or its related complication including NAFLD and NASH, calls for robust well designed studies better focusing the mechanisms controlling possible derangements of the numerous metabolic, toxic, and immunological actors participating to the gut-liver -axis. At present gut microbiota modulation by probiotics starts appearing as the most promising tool due to their safety, tolerability and efficacy, at least in the preliminary pilot studies hitherto published [34,45].

## Abbreviations

ALT: Alanine aminotransferase; DPP-4: Dipeptidyl peptidase-4; FFA: Free fatty acids; FXR: Farnesoid X receptor; GLP-1: Glucagon-like peptid-1; IR: Insulin resistance; NAFLD: Non alcoholic fatty liver disease; NASH: Non alcoholic steatohepatitis; PAMPs: Pathogen-associated molecular patterns; PUFAs: Polyunsaturated fatty acids; ROS: Reactive oxygen substances; SIBO: Small intestinal bacterial overgrowth; TLR: Toll-like receptor; UDCA: Ursodeoxycholic acid.

## Competing interests

Authors certificate that there are no conflict of interest.

Authors certificate that there are no financial competing interests.

## Authors’ contributions

PV and SL supervised the study and the final MS draft; GP, IDM, RDA prepared bibliographical background; CP, MS, and GP screened the papers retrieved by literature search. All worked on the draft MS and contributed to the text discussion. All authors read and approved the final manuscript.
